# Motivation of non-monogamous adults to engage in sex with their different partners

**DOI:** 10.3389/fpsyg.2022.961949

**Published:** 2022-09-21

**Authors:** Anna Kelberga (Kelberg), Baiba Martinsone

**Affiliations:** Department of Psychology, University of Latvia, Riga, Latvia

**Keywords:** sex, sexual motivation, non-monogamy, reasons for sex, reasons for sexual activity, sex with different partners

## Abstract

This study compared motivations of individuals in non-monogamous relationships to engage in sex with their different partners (*n* = 596, out of which 103 non-consensual non-monogamous, 135 polyamorous, 204 swinging, 154 in open relationships; women—38.8%, men—59.7%, other gender—1.5%; age range: from 18 to 65+ years; 86% of respondents between 25 and 54 years old; majority of the respondents are in a long-term relationship). The research aim was to identify whether there are differences in reasons to engage in sex with respondents’ primary versus secondary partners. Presented with 17 reasons to engage in sexual activity, the respondents rated the frequency with which they engage in sex for each reason with their different partners. Questions for 14 reasons to engage in sex were created based on the YSEX? questionnaire and three questions were created specifically for non-monogamous population. The three new questions addressed the desire for a specific type of sex (such as kink, fetish, etc.), desire to have sex with a partner of another gender than one’s primary partner, and desire to experience the thrill of the forbidden. The results indicated that there are significant differences in motivation to engage in sex with one’s primary versus secondary partner for most of the reasons. Additionally, this study investigated whether there are differences in motivation to engage in sex with different partners depending on the relationship arrangement. The data analysis shows that there are differences in reasons to engage in sex with one’s different partners between non-consensually and consensually non-monogamous groups. This research compliments existing body of research with new reasons to engage in sex, it demonstrates that non-monogamous people engage in sex with their different partners for different reasons and reveals that these may vary depending on the type of the relationship arrangement.

## Introduction

While research on non-monogamy is on the rise, it is still an understudied topic (Rubel and Bogaert, 2015). A substantial body of research studied relationship quality, satisfaction, well-being, health, happiness among people engaged in non-monogamous relationships, as well as stigma around these relationships (Conley et al., 2013; Rubel and Bogaert, 2015; Mogilski et al., 2017, 2020; Moors et al., 2017; Sizemore and Olmstead, 2017; Balzarini et al., 2019). Other studies investigated reasons to engage in sex among various populations (Meston and Buss, 2007; Wood et al., 2014; Armstrong and Reissing, 2015; Wyverkens et al., 2018; Kelberga and Martinsone, 2021). However, reasons to engage in sex with one’s various partners were not yet addressed.

### Non-monogamy

A non-monogamous relationship is a relationship, where one or more partners are not sexually exclusive to each other, whether there is or no explicit agreement between partners about sexual non-exclusivity. Monogamy and non-monogamy are not binary opposites, but rather a continuum along which relationships can be defined (Nelson, 2013; Parsons et al., 2013; Ferrer, 2018; Kelberga and Martinsone, 2021). Nelson (2013) invites to view monogamy and non-monogamy as a continuum that varies not only in its extent, but also can be seen through the prism of various facets, including, but not limited to, thoughts, fantasy, sex, love, flirtation, etc. Non-monogamous relationships are not a modern-day phenomenon and have existed in various forms throughout the history of the humanity (Ryan and Jetha, 2010; Zimmerman, 2012). Mogilski et al. (2017) argues that non-monogamous relationships occur in an array of forms across cultures, that includes serial monogamy – several consecutive mutually monogamous partners across the lifespan (Pinkerton and Abramson, 1993; Fisher, 2011), monogamish relationships – a relationship defined by a degree of openness to sexual and/or emotional relationships outside the primary dyad (Berry and Barker, 2014), polygyny – the marriage of one man to more than one woman, polyandry – the marriage of one woman to more than one man, polygynandry – a group marriage, non-consensual non-monogamy (NCNM) – secret sex with another partner/s (Conley et al., 2013; Rubel and Bogaert, 2015), consensual non-monogamy (CNM) – a relationship that has some degree of acceptance of extradyadic sexual and/or romantic relationships (Loue, 2006; Parsons et al., 2013). Some other authors propose relationship anarchy – intimate relationships characterized by anarchist principles such as autonomy, anti-hierarchical practices, lack of state control, anti-normativity, and community interdependence (Nordgren, 2012) and “nougamy” – a rejection of mono/poly binary (Ferrer, 2018). This study does not explore relationship forms that are illegal in the Western word, e.g., polygyny, polyandry, and polygynandry (Tucker, 2014), and focuses on most common forms of non-monogamy – infidelity (“non-consensual non-monogamy”) and consensual non-monogamy, represented by open relationships, swinging and polyamory. It is important to note, the above-mentioned categories are not mutually exclusive or collectively exhaustive and on the individual level definitions may be inaccurate due to subtle nuances of the complexity of human sexual relationships (Matsick et al., 2014).

For the purposes of this study a non-consensual non-monogamous relationship is defined as a relationship where one of the partners in a committed relationship is having a secret extradyadic sex with another partner or partners (Rubel and Bogaert, 2015). In contrast to consensually non-monogamous relationships, engaging in sexual intercourse with someone outside of the relationship without their consent is associated with the feelings of betrayal and loss of trust when the cheating partner’s engagement with someone else is being exposed (Rubel and Bogaert, 2015; Schnarre and Adam, 2017) and is a common cause for separation or divorce (Amato and Previti, 2003). It is hard to estimate the prevalence of secretive extradyadic sex, mainly due to stigma and potential devastating consequences of exposure, but also due to used methodology, the sample surveyed, and the definition of infidelity used by the researchers. For example, Vowels et al. (2022) eloquently summarize the problem of definition of infidelity – infidelity can be defined as engaging in sexual or emotional relationship outside the dyad, it may include various behaviors from intercourse to an emotional connection, flirting, pornography, sexting or watching life webcam porn. Thus, scientific literature presents a wide range of infidelity prevalence – anywhere from 26 to 76% (Emmers-Sommer et al., 2010; Brandon, 2011; Zimmerman, 2012; Conley et al., 2013; Thompson and O’Sullivan, 2016).

A relationship that explicitly allows for extradyadic romantic or sexual relationships is called consensually non-monogamous (Conley et al., 2013; Burleigh et al., 2017). Barker and Langdridge (2010) point out that consensually non-monogamous partners agree on arrangements which might include ongoing, emotionally committed relationships and short-term sexual adventures. It is estimated that up to 5% of Americans (Rubin et al., 2014), 2.5% of Canadians (Fairbrother et al., 2019) and around 3% of Norwegians (Træen and Thuen, 2022) are engaged in consensual non-monogamy at an any given time and around a quarter of the Americans, Canadians and Norwegians at least once have engaged in a consensually non-monogamous relationship in their lifetime (Fairbrother et al., 2019; Rubel and Burleigh, 2020; Træen and Thuen, 2022). The most studied and prevalent forms of consensual non-monogamy are polyamory, swinging, and open relationships (Richards and Barker, 2013; Rubel and Bogaert, 2015). In a swinging relationship a couple would engage in sexual activities with people other than their primary partner, typically at a party or in another social setting (Matsick et al., 2014). Polyamorous relationships are those in which people experience both sexual and emotional relationships with multiple partners concurrently (Matsick et al., 2014; Grunt-Mejer and Cambell, 2016). Contrary to swingers, polyamorous people are more likely to describe their multiple relationships as having a romantic and emotional component, rather than being strictly sexual (Sheff and Hammers, 2011, as mentioned in Matsick et al., 2014). An open relationship is an arrangement where either one or both partners seek sexual relationships independently from one another (in contrast to swingers, who may pursue extra-dyadic sexual relationships in the presence of their partners and in contrast to polyamorous people who would usually emphasize an emotional connection with their partner; Matsick et al., 2014). While above-described taxonomy is convenient both for researchers and general population, it may not be fully accurate and nuanced when applied to individual relationships (Matsick et al., 2014) and should be perceived as a general trend.

### Various partners of non-monogamous individuals

Generally, a non-monogamous arrangement implies that a non-monogamous individual is involved with more than one partner. While a relationship arrangement defines the relationship’s structure and the degree of secrecy/transparency about relationships with all involved parties, in the following paragraphs the focus is on the partners’ subjective status relative to one another. The two most common relationship configurations are the primary/secondary model and the multiple primary partners model (or equal partners model).

The primary/secondary partner model is the most practiced arrangement (Barker, 2005; Labriola, 2010). In this configuration, participants of a primary relationship assign a subjectively higher status to each other compared to other partners (Labriola, 2010; Balzarini et al., 2017). A primary dyad represents a primary relationship unit, and this couple would usually live together and share finances, while other relationships would receive less priority and therefore, less time and other resources (Labriola, 2010; Balzarini et al., 2017). Usually, there is an explicit or implicit agreement that no outside relationship is allowed to become equally or more important compared to the primary partnership (Labriola, 2010). Other partners beyond the primary relationship are often referred to as non-primary partners or ‘secondary’ partners (Balzarini et al., 2017). A secondary relationship often consists of partners who live in separate households, do not share finances (Klesse, 2006), are afforded relatively less time, energy, and priority in a person’s life than are primary partners (Balzarini et al., 2017). Furthermore, a secondary relationship often consists of less ongoing commitments, such as future plans together (Veaux et al., 2014). Some couples would pick a secondary partner together to have casual sex with (Labriola, 2010). Other couples would allow each other to independently have outside sexual relationships with secondary partners, either casual or long-term (Labriola, 2010).

The multiple primary partners model includes three or more people in a primary relationship in which all members are equal partners (sometimes also called “polyfidelitous”). Instead of a couple having priority and control in the relationship, in this arrangement all relationships are viewed as equal and primary or have a potential of becoming primary and each of the partners would have equal power to negotiate for what they want in terms of time, commitment, living and financial arrangements, sex, and other needs (Labriola, 2010). The Multiple primary partners model may also be presented in an altered form – when an individual remains single but participates in more than one relationship without the constraints of a primary relationship (multiple non-primary model; Labriola, 2010). By and large, this is an individual’s subjective decision to assign a certain status to their multiple partners - either hierarchical (primary/secondary) or not (equal). A couple might make an explicit agreement about being primary to each other, also to being mutually primary or asymmetrically primary, where one of the partners considers the other partner as primary, but not vice a versa.

### Research aims

The present study examines differences of sexual motives to engage in sex with different partners of non-monogamous individuals. The reasons why humans engage in sex are numerous and compound, and go far beyond the obvious pleasure, procreation, and relief of sexual tension. While there were some earlier studies that attempted to expand the list of reasons to engage in sex (see Leigh, 1989; Hill and Preston, 1996), the most extensive one was presented by Meston and Buss (2007) and included about one and a half hundred reasons to engage in sex (later these were grouped into four overarching factors and 13 subfactors). While the original YSEX? questionnaire (Meston and Buss, 2007) was developed using mostly heterosexual monogamous college students as a sample group, it was at the foundation of several other studies that have explored sexual motivations of various other populations. Since the development of YSEX? (Meston and Buss, 2007) other studies investigated how these reasons change under different circumstances (see Armstrong and Reissing, 2015, for women’s motivations to have sex in casual and committed relationships with male and female partners; Wood et al., 2014, for reasons for having sex among lesbian, bisexual, queer, and questioning women in romantic relationships; Wyverkens et al., 2018, for a replication study in different age groups, and Kelberga and Martinsone (2021), for difference in motivation to engage in sex among monogamous and non-monogamous respondents). Previous research also suggests that humans may practice strategic pluralistic mating strategy and engage in sex to fulfill different relationship needs (Gangestad and Simpson, 2000; Mogilski et al., 2017). This applies to all genders and to people both in consensually and non-consensually non-monogamous relationships (Mogilski et al., 2017).

While in the last decade the topic of non-monogamy has drawn much attention of the scientific and general communities, it is still understudied. Only a few studies have investigated motivation to engage in sex among certain groups of non-monogamous people (Wood et al., 2018; Mitchell et al., 2020), but current scientific knowledge on the reasons to engage in sex with one’s various partners is especially insufficient. Investigating the reasons to engage in sex with one’s primary and secondary partner allows to get a better understanding whether there are differences in motivation to have sex with different partners.

It was hypothesized that individuals would report similar levels of motivation to engage in sex with their different partners motivated by their physical desirability and to experience sexual pleasure. Previous research proved physical desirability and pleasure to be the most frequent and similarly motivating reasons to engage in sex for both monogamous and non-monogamous individuals (Kelberga and Martinsone, 2021) and different age groups (Wyverkens et al., 2018). However, as different partners fulfill different relationship needs (Gangestad and Simpson, 2000; Mogilski et al., 2017; Mitchell et al., 2020), it was hypothesized that the respondents would report different levels of motivation to engage in sex with their different partners for all other reasons. For example, it was expected that individuals would seek sex with their primary partners more often to procreate, for utilitarian reasons, to express love and commitment, or other feelings like anger or feeling sorry. However, individuals would more often engage in sex with their secondary partners seeking new experiences, a different type of sex (Carlström and Andersson, 2019; Vilkin and Sprott, 2021), or another gender (Jordal, 2011; Compton and Bowman, 2017; Shao et al., 2021), and to experience the thrill of the forbidden (Morin, 1996; Fishbach, 2009; Ruedy et al., 2013).

While there are several forms of non-monogamous relationship arrangements, the prevailing two larger groups are non-consensual non-monogamy and consensual non-monogamy. The main distinctive feature of these two types of relationships is whether partners engage in relationships with other partners with a degree of consent from another partner or in secret, with transparency and honesty being high on the value chain for consensually non-monogamous population. Thus, another goal of this study was to explore whether there are differences in reasons to engage in sex with one’s primary and secondary partners between consensually non-monogamous (CNM) and non-consensually non-monogamous groups.

## Materials and methods

### Participants

The target population was defined as individuals of legal age (18 years or older) in non-monogamous relationships (either married or in committed relationships to one of the partners). A simple random sampling technique was used to collect data online. Invitation to participate in a survey was published on social media websites, forums and websites for people with specific interests (e.g., swinging). A total of 596 non-monogamous respondents have completed the survey (out of which 103 were non-consensual non-monogamous, 135 polyamorous, 204 swinging, 154 in open relationships; women—38.8%, men—59.7%, other gender—1.5%). Age distribution of the study participants: 8.2% were between 18 and 24 years old, 39.9% were between 25 and 34 years old, 29.7% between 35 and 44, 16.4% between 45 and 54, 4.95% between 55 and 64 and 0.8% older than 65 years old. See Table 1. for a detailed breakdown of the respondents’ relationship arrangements, gender, sexual self-identification, sexual attraction and sexual behavior.

**Table 1:** Gender, sexual self-identification, attraction and behavior across the relationship arrangement groups.

				Sexual self-identification				Sexual attraction						Sexual behavior		
Relationship arrangement	Self-identified gender	Total number of respondents in this cathegory of relationship arrangement	% of total respondents in this cathegory of relationship arrangement	Other Sexual orientation, %	Heterosexual, %	Gay/ Lesbian, %	Bisexual, %	Only attracted to females, %	Mostly attracted to females, %	Equally to males and females, %	Mostly attracted to males, %	Only attracted to male, %s	Not sure, %	Men only, %	Women only, %	Both men and women, %
Non-consensually non-monogamous	Females	32	31.1	0.0	78.1	0.0	21.9	3.1	0.0	0.0	78.1	18.8	0.0	68.8	0.0	31.3
	Males	71	68.9	1.4	84.5	0.0	14.1	69.0	28.2	1.4	1.4	0.0	0.0	0.0	80.3	19.7
Polyamorous	Females	69	51.1	15.9	29.0	4.3	50.7	1.4	8.7	30.4	37.7	18.8	2.9	31.9	4.3	63.8
	Males	59	43.7	1.7	79.7	5.1	13.6	57.6	28.8	6.8	3.4	3.4	0.0	5.1	71.2	23.7
Swingers	Females	71	34.8	7.0	56.3	1.4	35.2	1.4	4.2	15.5	62.0	16.9	0.0	26.8	0.0	73.2
	Males	133	65.2	5.3	72.9	0.0	21.8	62.4	29.3	6.8	1.5	0.0	0.0	1.5	72.9	25.6
Open relationships	Females	59	38.3	1.7	50.8	5.1	42.4	3.4	5.1	18.6	49.2	23.7	0.0	40.7	3.4	55.9
	Males	93	60.4	2.2	72.0	2.2	23.7	51.6	35.5	9.7	1.1	2.2	0.0	3.2	75.3	21.5

### Measures

To identify a relationship arrangement practiced by respondents the following multiple choice options were presented to the study participants. These items were presented in random order. The sources of the definitions are referenced in the brackets but were not included in the survey.

1. In a non-consensual non-monogamous relationship, i.e., having a partner while having secret sex with another partner/s (Conley et al., 2013; Rubel and Bogaert, 2015).
2. Polyamorous, i.e., being engaged in relationships in which not only sexual but emotional relationships are conducted with multiple partners (Grunt-Mejer and Cambell, 2016).
3. Swinging, i.e., in a relationship in which a couple engages in sex with other partners outside of a committed relationship, usually at parties or social situations where both partners are present (Grunt-Mejer and Cambell, 2016).
4. In an open relationship, i.e., being in a relationship in which couple explicitly agrees that partners can have other sex partners outside of a committed relationship (Rubel and Bogaert, 2015; Grunt-Mejer and Cambell, 2016).

To investigate reasons to engage in sex the authors of this study developed a questionnaire that included 14 overarching questions adopted from YSEX? questionnaire (Meston and Buss, 2007) and three additional questions to address the needs of non-monogamous respondents. As the original YSEX? questionnaire (Meston and Buss, 2007) includes 142 items, it was time consuming to answer the whole set of questions about multiple partners and would reduce the completion rate (Saleh and Bista, 2017). However, as the questions of the original YSEX? questionnaire is grouped into 4 factors and 13 subfactors, one question was formulated to represent each of the subfactors. “Resources” subfactor was represented by two items – “resources” (such as promotion, money, etc.) and “procreation” as the authors of the study reasoned that it would be beneficial to separate as important reason as procreation. This resulted in a following set of questions:

1. Stress reduction: I wanted to release stress, anxiety, tension or to fight boredom
2. Pleasure: I was sexually aroused or wanted to experience physical pleasure
3. Physical desirability: The person was physically attractive
4. Experience seeking: I wanted new sexual experience or to act out a fantasy
5. Resources: I wanted to get resources from that person (such as promotion, money, etc.)
6. Procreation: I wanted to conceive a child
7. Social status: I wanted to enhance my social status or reputation
8. Revenge: I wanted my partner to feel jealous or hurt
9. Utilitarian reasons: I had sex for utilitarian reasons (such as burning calories, hoping to get rid of a headache or keeping warm)
10. Love and commitment: I wanted to feel connected to the person, express my love and commitment
11. Expression: I wanted to have sex in order to express my feelings such as being sorry, thankful, etc.
12. Self-esteem boost: I wanted to boost my self-esteem (such as feeling attractive or powerful)
13. Duty/pressure: I felt obligated or did not know how to say “no”
14. Mate guarding: I wanted to keep my partner from having sex with someone else.

YSEX? questionnaire (Meston and Buss, 2007) was created based on the responses of heterosexual monogamous college students. However, non-monogamous individuals often seek multiple relationships to satisfy their diverse needs (Mitchell et al., 2014; Balzarini and Muise, 2020) and thus this population may have other needs than monogamous individuals, which resulted in additional items to engage in sex. Consequently, another three reasons to engage in sex were added to the final questionnaire. Since some authors (Carlström and Andersson, 2019; Vilkin and Sprott, 2021) suggest that non-conventional sexual practices like BDSM (bondage and discipline, dominance and submission, and sadism and masochism) or kink might be a significant factor to engage in a consensually non-monogamous relationship, the following questionnaire item was developed:

15. Specific sex: “I wanted to have sex which I cannot have with my other partner (such as kink, fetish, anal, etc.)”

Some authors point out that some couples “open up” to address their bisexual needs (see Jordal, 2011; Compton and Bowman, 2017). This idea is supported by Mogilski et al. (2017) who suggest that multi-partnered individuals are more likely to describe their sexuality in non-polar ways compared to monogamous individuals. Shao et al. (2021) explore “mixed orientation marriages,” where one of the partners is heterosexual and the other is not (i.e., bisexual). To address the needs of these groups, the following item was added:

16. Another gender: “I wanted to have sex with a person of an opposite gender than my other partner.”

The last item of the questionnaire was developed based on the idea that sometimes an unethical behavior can trigger positive affect on the cheating person (Ruedy et al., 2013). Taboo experiences might be more attractive than those that are not prohibited (Fishbach, 2009; Ruedy et al., 2013) and might explain the “cheater’s high” or the pleasure of the thrill of the forbidden. Morin (1996) states that violation of prohibitions (e.g., engaging with someone with whom one is not supposed to, or undergoing a risk of discovery) might have high potential for eroticism and arousal. This resulted in the following question:

17. Thrill of the forbidden: “I wanted to experience the thrill of doing something forbidden.”

As the original YSEX? questionnaire (Meston and Buss, 2007) was modified and enhanced, a factor analysis was carried out and the results have shown that each variable is loading heavily on a single factor only. This indicates that each question is measuring a different dimension (latent trait), both for the results of the first partner and the second partner.

Answers to the survey questions were given on a Likert scale from 1 to 5, where “1” was “none of my experiences” and “5” was “all of my experiences.”

To compare two groups of partners (i.e., a group of first partners and a group of second partners) of non-monogamous respondents the authors used Wilcoxon Z test.

To find out if there are differences in reasons to engage in sex with one’s various partners between two groups – consensually and non-consensually non-monogamous individuals – the mean differences scores between both partners of consensually and non-consensually non-monogamous individuals were compared (MANOVA, fixed factor – relationship arrangement).

### Procedure

Before publishing the survey questions online, the first author of this paper conducted four pilot interviews over video call and in person with one couple and three individuals who were at the time of the interview in a non-monogamous relationship (one engaged in non-consensual non-monogamous relationship, one swinging couple, one solo-polyamorous and one engaged in an open relationship). Interviews were semi-structured and asked the respondents about their motivation to engage in sex with their different partners. The answers were transcribed in a note form. After these interviews a questionnaire was finalized and hosted on a SurveyMonkey website. The survey consisted of an informed consent form, demographic questions, a multiple-choice question about relationship arrangement, the core questions and an optional field for contact information should the participants want to answer further questions.

The invitation to participate in the survey was posted online on 24 different websites: on reddit.com in 42 different subreddits, on various dating forums (including websites targeting specifically elderly people, swingers, polyamorous people, specific geographical locations, and hook ups), discussion groups (marriage advice, confessions) and social media.

### Data analytic plan

Following descriptive statistics of the main measures and ranking of reasons in the order of reported frequency, the theoretical model was tested. To identify similar and different levels of motivation to engage in sex with different partners, the group of first partners and the group of second partners were compared using Wilcoxon Z test. Then, a deeper look was taken at the level of relationship arrangement. As motivation to engage in sex with various partners may be different depending on whether the individual is engaged in consensually or non-consensually non-monogamous relationship, MANOVA was used to assess differences between groups.

Ethical approval for the study was granted by Ethics Committee for Humanities and Social Sciences research involving human participants, University of Latvia.

## Results

### Non-monogamous group

To answer the research question if there are any differences in motivation to engage in sex with one’s various partners, self-reported frequencies to engage in sex for various reasons with different partners were calculated and compared (see Table 2.).

**Table 2:** Reasons of non-monogamous respondents to engage in sex their different partners.

Reasons to Engage in Sex	Non-monogamous Respondents’ Partner 1			Non-monogamous Respondents’ Partner 2			
	*n* = 596			*n* = 596			
	M	SD	Mdn	M	SD	Mdn	Z
Stress reduction	2.68	1.005	3	2.40	1.276	2	−**5.950** ^***^
Pleasure	3.97	0.728	4	3.83	1.059	4	−**3.043** ^**^
Physical desirability	3.84	0.973	4	3.72	1.113	4	−1.933
Experience seeking	2.92	0.967	3	3.34	1.176	4	−**7.261** ^***^
Resources	1.54	1.047	1	1.53	1.069	1	−0.183
Procreation	1.88	1.141	1	1.41	0.963	1	−**10.355** ^***^
Social status	1.55	1.048	1	1.55	1.017	1	−0.074
Revenge	1.46	0.980	1	1.48	1.007	1	−0.843
Utilitarian	1.80	1.061	1	1.62	1.081	1	−**5.911** ^***^
Love and commitment	3.92	0.851	4	2.55	1.421	2	−**15.612** ^***^
Expression	2.70	1.110	3	1.97	1.219	1	−**12.290** ^***^
Self-esteem boost	2.57	1.135	3	2.69	1.282	3	−**2.683** ^**^
Duty/pressure	1.91	1.019	2	1.71	1.040	1	−**5.155** ^***^
Mate guarding	1.65	1.091	1	1.55	1.040	1	−**3.029** ^**^
Specific sex	1.94	1.154	1	2.48	1.354	2	−**8.269** ^***^
Another gender	1.73	1.107	1	1.97	1.265	1	−**5.446** ^***^
Thrill of the forbidden	2.12	1.108	2	2.71	1.323	3	−**9.709** ^***^

Significant differences are highlighted in bold.

* *p* < 0.05;

** *p *< 0.01;

*** *p* < 0.001.

Respondents were prompted to assign status to their partners – either “the primary/secondary” or “the multiple primary partners model/equal.” Across all presented relationship arrangements (non-consensually non-monogamous, polyamorous, swinging, and open relationships) majority (73%) of the study respondents indicated that their relationship is best described by primary/secondary model where one of the partners has a higher importance and status compared to respondent’s another partner. 83% of the cheating respondents viewed one of their partners as primary, 76% of swinging, 71% in an open relationship and 61% in polyamorous relationships. Moreover, when asked about commitment levels to each of the partners (higher level of commitment is a trait of primary relationship, Balzarini et al., 2017), most respondents in the multiple primary partners model/equal relationship configuration showed difference in commitment, being skewed towards the first partner. Data analysis was performed twice – with and without respondents who indicated that their partners are equal in status. This analysis did not show any significant differences from the results of the study. While a larger sample of respondents in a relationship with equal partners in their status potentially could impact the results of the study, for the purposes of this study all partners are divided into primary and secondary.

Desire to experience physical pleasure is the most frequent reason to engage in sex with both primary and secondary partners, however, respondents have engaged in sex for this reason slightly more often with their primary partners than with their secondary partners (Z = −3.043, *p* < 0.01; see Table 3. for the reasons to engage in sex in the order of reported frequency among non-monogamous individuals and Table 2. for reasons of non-monogamous respondents to engage in sex their different partners).

**Table 3:** Reasons to engage in sex in the order of reported frequency among non-monogamous individuals.

Reasons to Engage in Sex	Non-monogamous Respondents’ Partner 1			Reasons to Engage in Sex	Non-monogamous Respondents’ Partner 2		
	*n* = 596				*n* = 596		
	M	SD	Mdn		M	SD	Mdn
Pleasure	3.97	0.728	4	Pleasure	3.83	1.059	4
Love and commitment	3.92	0.851	4	Physical desirability	3.72	1.113	4
Physical desirability	3.84	0.973	4	Experience seeking	3.34	1.176	4
Experience seeking	2.92	0.967	3	Thrill of the forbidden	2.71	1.323	3
Expression	2.70	1.110	3	Self-esteem boost	2.69	1.282	3
Stress reduction	2.68	1.005	3	Love and commitment	2.55	1.421	2
Self-esteem boost	2.57	1.135	3	Specific sex	2.48	1.354	2
Thrill of the forbidden	2.12	1.108	2	Stress reduction	2.40	1.276	2
Specific sex	1.94	1.154	1	Expression	1.97	1.219	1
Duty/pressure	1.91	1.019	2	Another gender	1.97	1.265	1
Procreation	1.88	1.141	1	Duty/pressure	1.71	1.040	1
Utilitarian	1.80	1.061	1	Utilitarian	1.62	1.081	1
Another gender	1.73	1.107	1	Social status	1.55	1.017	1
Mate guarding	1.65	1.091	1	Mate guarding	1.55	1.040	1
Social status	1.55	1.048	1	Resources	1.53	1.069	1
Resources	1.54	1.047	1	Revenge	1.48	1.007	1
Revenge	1.46	0.980	1	Procreation	1.41	0.963	1

Physical desirability is the second most frequent on the list to engage in sex with secondary partners and the third on the list for reasons to engage in sex with primary partners, not showing any statistically significant difference between these two groups.

To express love and commitment is the second most frequent reason to engage in sex with one’s primary partner and only number six on the list with one’s secondary partner – respondents reported engaging in sex to express love and commitment with their primary partners more often than with their secondary partners (Z = −15.612, *p* < 0.001).

Experience seeking is the third most frequent reason to engage in sex with one’s secondary partner, which is significantly more often than with one’s primary partner (Z = −7.261, *p* < 0.001).

The fourth most frequent reason to engage in sex with one’s secondary partner is the thrill of the forbidden, a newly added reason to the list of the reasons to engage in sex. This reason is number eight with primary partners (Z = −9.709, *p* < 0.001).

Expression of feelings like being sorry or thankful is fifth most frequent reason to engage in sex with one’s primary partner and ninth with secondary partner, indicating that individuals engage in sex for this reason significantly more often with their primary partners (Z = −12.290, *p* < 0.001).

Respondents reported on relying on their primary partners more than their secondary partners to reduce their stress levels with the help of sex (Z = −5.950, *p* < 0.001). Meanwhile, they rely on their secondary partners more often than on their primary partners to boost their self-esteem through sexual activity (Z = −2.683, *p* < 0.001).

Specific type of sex, which one cannot have with their other partner (such as kink, fetish, anal, etc.) is a more common motivation to engage in sex with one’s secondary partner than primary partner (Z = −8.269, *p* < 0.001). This was a newly added question to the original questionnaire and proved to be an important motivator.

Sex out of duty or pressure are at a similar position in the hierarchy of reasons to engage in sex both with primary and secondary partners, but respondents reported to engage in sex out of duty or pressure more often with their primary partners than secondary partners (Z = −5.155, *p* < 0.001).

Mate guarding is a relatively low motivator to engage in sex both with primary and secondary partners. Respondents reported that they have engaged in sex with their primary partners to keep them from having sex with someone else significantly more often than they have engaged in sex for the same reason with their secondary partner (Z = −3.029, *p* < 0.001).

Another reason to engage in sex is the desire to have sex with a person of the opposite gender than one’s another partner. This reason is more pronounced with secondary partners (Z = −5.446, *p* < 0.001).

Respondents reported engaging in sex with their primary partners more often than with their secondary partners for utilitarian reasons, such as burning calories, hoping to get rid of a headache or keeping warm (Z = −5.911, *p* < 0.001).

Procreation is the least often reason to engage in sex with secondary partners: study participants reported to engage in sex to conceive a child significantly more often with their primary partners (Z = −10.355, *p* < 0.001).

The respondents engaged in sex least frequently for the following three reasons - to enhance their social status or reputation, to get resources from a person (such as promotion, money, etc.) and out of revenge (to make their other partner feel jealous or hurt). These reasons are similarly unpopular reasons to engage in sex both with primary and secondary partners and showed no statistical differences between groups.

### Consensually vs. non-consensually non-monogamous individuals

To answer the second research question if there are any differences in motivation to engage in sex with one’s various partners, depending on whether an individual is in a consensually or a non-consensually non-monogamous relationship, self-reported frequencies to engage in sex for various reasons with different partners were calculated and compared (see Table 4 for differences, Tables 5, 6 for the rankings of reasons in the order of reported frequency).

**Table 4:** MANOVA for differences in scores for reasons to engage in sex between primary and secondary partners among non-consensually and consensually non-monogamous individuals.

Reasons to Engage in Sex	Non-consensually non-monogamous respondents		Consensually non-monogamous respondents		
	*n* = 103		*n* = 493		
	M (difference in scores between P1 and P2)	SD	M (difference in scores between P1 and P2)	SD	MANOVA value of p
Stress reduction	−0.16	1.430	0.43	1.172	**0.000** ^***^
Pleasure	−0.10	1.256	0.16	1.064	0.051
Physical desirability	−0.24	1.414	0.16	1.269	**0.010** ^**^
Experience seeking	−1.22	1.545	−0.39	1.361	**0.000** ^***^
Resources	0.08	0.843	0.02	0.684	0.500
Procreation	0.63	1.148	0.54	0.919	0.445
Social status	−0.06	0.757	−0.03	0.673	0.699
Revenge	−0.14	0.706	−0.03	0.529	0.116
Utilitarian	0.20	0.733	0.22	0.737	0.785
Love and commitment	1.26	1.867	1.90	1.522	**0.001** ^***^
Expression	0.70	1.511	1.00	1.203	**0.047** ^*^
Self-esteem boost	−0.60	1.161	−0.10	1.147	**0.000** ^***^
Duty/pressure	0.43	1.174	0.18	0.840	**0.026** ^*^
Mate guarding	0.02	1.062	0.12	0.718	0.324
Specific sex	−1.59	1.843	−0.48	1.373	**0.000** ^***^
Another gender	−0.19	0.861	−0.36	1.140	0.186
Thrill of the forbidden	−1.44	1.606	−0.64	1.314	**0.000** ^***^

Significant differences are highlighted in bold.

* *p* < 0.05;

** *p* < 0.01;

*** *p* < 0.001.

**Table 5:** Reasons to engage in sex in the order of reported frequency among non-consensually non-monogamous individuals (NCNM).

Reasons to Engage in Sex	NCNM Respondents’ Partner 1			Reasons to Engage in Sex	NCNM Respondents’ Partner 2		
	*n* = 103				*n* = 103		
	M	SD	Mdn		M	SD	Mdn
Pleasure	3.91	0.794	4	Pleasure	3.98	1.038	4
Love and commitment	3.76	0.923	4	Physical desirability	3.89	1.009	4
Physical desirability	3.66	1.044	4	Experience seeking	3.64	1.128	4
Stress reduction	2.68	1.104	3	Thrill of the forbidden	3.27	1.388	4
Experience seeking	2.62	1.077	3	Specific sex	3.17	1.463	4
Expression	2.55	1.152	3	Self-esteem boost	3.01	1.283	3
Self-esteem boost	2.49	1.128	2	Stress reduction	2.83	1.314	3
Duty/pressure	2.15	1.150	2	Love and commitment	2.74	1.488	3
Thrill of the forbidden	2.14	1.268	2	Expression	1.98	1.244	1
Procreation	2.00	1.155	2	Another gender	1.77	1.222	1
Specific sex	1.89	1.196	1	Duty/pressure	1.69	1.039	1
Utilitarian	1.75	1.118	1	Mate guarding	1.66	0.996	1
Mate guarding	1.71	1.117	1	Utilitarian	1.60	1.166	1
Another gender	1.55	1.109	1	Social status	1.53	1.46	1
Resources	1.53	1.065	1	Resources	1.52	1.119	1
Social status	1.49	1.028	1	Procreation	1.46	1.027	1
Revenge	1.38	0.930	1	Revenge	1.46	1.008	1

**Table 6:** Reasons to engage in sex in the order of reported frequency among consensually non-monogamous individuals (CNM).

Reasons to Engage in Sex	CNM Respondents’ Partner 1			Reasons to Engage in Sex	CNM Respondents’ Partner 2		
	*n* = 493				*n* = 493		
	M	SD	Mdn		M	SD	Mdn
Pleasure	3.98	0.714	4	Pleasure	3.80	1.062	4
Love and commitment	3.96	0.831	4	Physical desirability	3.69	1.131	4
Physical desirability	3.87	0.954	4	Experience seeking	3.28	1.177	3
Experience seeking	2.98	0.931	3	Self-esteem boost	2.63	1.273	3
Expression	2.73	1.099	3	Thrill of the Forbidden	2.59	1.279	3
Stress reduction	2.68	0.985	3	Love and Commitment	2.51	1.405	2
Self-esteem boost	2.58	1.137	3	Specific sex	2.34	1.286	2
Thrill of the forbidden	2.11	1.073	2	Stress reduction	2.31	1.250	2
Specific sex	1.95	1.146	2	Another gender	2.01	1.270	1
Duty/pressure	1.86	0.983	2	Expression	1.97	1.214	1
Procreation	1.85	1.138	1	Duty/pressure	1.72	1.041	1
Utilitarian	1.81	1.050	1	Utilitarian	1.62	1.063	1
Another gender	1.77	1.103	1	Social status	1.56	1.012	1
Mate guarding	1.63	1.086	1	Resources	1.53	1.060	1
Social status	1.57	1.052	1	Mate guarding	1.53	1.049	1
Resources	1.55	1.044	1	Revenge	1.49	1.007	1
Revenge	1.47	0.991	1	Procreation	1.40	0.950	1

Non-consensually non-monogamous (NCNM) respondents reported engaging in sex with their secondary partners more often than with their primary partners to release stress compared to CNM respondents, who reported engaging in sex with their primary partner more often than secondary for this reason (Mn-CNM = −0.16. MCNM =0.43, *p* < 0.001).

Also, NCNM group reported engaging in sex with their secondary partners more often than with their primary partners due to their partner’s physical desirability compared to CNM group who reported engaging in sex with their primary partner more often than secondary motivated by their physical desirability (Mn-CNM = −0.24. MCNM =0.16, *p* < 0.01).

While both NCNM and CNM respondents reported engaging in sex with their secondary partners more often than with their primary partners for the following reasons – experience seeking, self-esteem boost, specific sex (kink, anal, etc.), and the thrill of the forbidden – NCNM reported to do it for these reasons significantly more often compared to CNM respondents (see Table 4 for details).

Both groups reported engaging in sex with their primary partners more often than with their secondary partners to feel connected, express their love and commitment and to express their other feelings (feeling sorry, thankful, etc.), but CNM group reported to do it for these reasons significantly more often compared to NCNM respondents (see Table 4 for details).

Last, but not the least, NCNM respondents reported to engage in sex with their primary partners out of duty significantly more often than CNM respondents (MNCNM =0.43. MCNM =0.18, *p* < 0. 05).

## Discussion and implications

The purpose of this study was to investigate motivation of non-monogamous adults to engage in sex with their different partners and to explore whether the reasons are different depending on whether an individual is in a consensually or non-consensually non-monogamous relationship. This study provided a better understanding of the reasons to engage in sex with different partners of non-monogamous individuals by adding three additional reasons to engage in sex, which proved to be significant motivators. This study found that there are significant differences in most of the reasons to engage in sex with one’s different partners, this way demonstrating that different relationships potentially carry different functions in one’s life. There are significant differences in reasons to engage in sex with one’s primary and secondary partners, except for three reasons – obtaining resources, social status enhancement and revenge – these are the most unpopular motivators to engage in sex in both groups and also showed no significant difference between primary and secondary partners and generally play little role in one’s motivation to engage in sex with any of the partners.

The study demonstrated that pleasure is the top reason to engage in sex both with one’s primary partner and one’s secondary partner. It is also the top reason to engage in sex for monogamous individuals (Kelberga and Martinsone, 2021), college students (Meston and Buss, 2007) and elderly individuals (Wyverkens et al., 2018).

The next top reason to engage in sex – physical desirability – is complicated. It is one of the top reasons to engage in sex for all groups previously investigated. CNM respondents seek their primary partners more often than secondary to engage in sex for this reason, while non-consensually non-monogamous – their secondary partner. A further investigation that considers various factors, including whether the non-monogamy status is symmetrical or asymmetrical within the partnership, is necessary to explain this difference.

Non-monogamous individuals would engage in sex with their secondary partners more often than with their primary partners seeking novelty (experience seeking), to boost their self-esteem or find what they cannot have in their primary relationship – specific type of sex that they do not engage in with their primary partner (such as kink, fetish, anal, etc.), another gender (to have sex with a person of an opposite gender than their primary partner) or to experience the thrill of the forbidden. To avoid misinterpretation, it is important to mention that experience seeking is not reserved to secondary partners – actually, it is very high on the list of reasons to engage in sex both with primary and secondary partners. Experience seeking is the most frequent reason to engage in sex after pleasure, physical desirability (and expressing love and commitment to a primary partner) and is more pronounced in relation to secondary partners as it is probably just easier to find novelty with a secondary partner than with the primary, since primary relationships tend to be longer in duration. Self-esteem boost is a more pronounced reason to engage in sex with one’s secondary partner rather than primary partner, especially for the NCNM group. If self-esteem is an issue, failure to secure a new partner might feel as a difficult topic to discuss with one’s primary partner and may stand behind these findings. Mate guarding was relatively low on the list of reasons to engage in sex both for non-monogamous respondents as a larger group and both CNM and NCNM groups separately. This finding goes in line with previous research (Mogilski et al., 2017) and expands it to non-monogamous population.

There are two reasons that stood out on the relationship arrangement level. First, NCNM group reported relying on their secondary partners more often than primary partners to relieve stress, while CNM group reported relying on their primary partners more often to deal with anxiety and stress. Sex is a potent instrument to reduce stress. However, previous studies have shown that sex has capacity to relieve stress in satisfying relationships, but not in unsatisfying relationships (Ein-Dor and Hirschberger, 2012). Additional research is needed to understand whether these differences may be explained with relationship satisfaction or any other factors. Second reason that changes depending on the relationship arrangement is physical desirability. CNM respondents report engaging in sex more often with their primary partners than secondary motivated by their physical desirability, while NCNM respondents – with their secondary partners. Again, more research is needed to understand underlying factors of this finding.

For all other reasons people would engage in sex more often with their primary partner. People would engage in sex with one’s primary partner more often than with their secondary partner both to express love and commitment and other feelings, such as being sorry or thankful. Mogilski et al. (2020) state that primary relationships tend to be longer in duration and non-monogamous individuals tend to be selective with whom they maintain long-term relationships, thus higher rates of emotional expression of love and commitment to the primary partner seems natural. Procreation is significantly more often reserved to primary partners, which may be explained by the more significant investment people tend to make in their primary relationships (Buss et al., 2017). However, in line with the dual mating strategy hypothesis, that postulates that people may engage in sex with other partners than one’s primary partner to obtain good genes (Gangestad and Haselton, 2015), a number of respondents reported engaging in sex with their secondary partner to conceive a child. Utilitarian reasons, duty and pressure and mate-guarding lead to sex with primary partners more often than with secondary.

### Limitations and future directions

This study managed to gather responses of a large international and diversified sample of non-monogamous respondents. This group is difficult to reach, and, in this study, the non-monogamous sample is represented by people in different relationship arrangements, including non-consensually non-monogamous adults, swingers, polyamorous adults, and adults in open relationships.

To assess the motivation of respondents to engage in sex, in line with previous practice, this study used a questionnaire that measured subjective frequency of engagement in sex with their partners. However, frequency may not be the best way to research motivation, especially in the context of non-monogamous relationships.

Authors believe that an additional question should have been added to the questionnaire to better understand motivation to engage in sex. That question should reflect an individual’s desire to have multiple partners to minimize the risk of depending on one partner and having a “backup” option. This is supported by the mate switching hypothesis by Buss et al. (2017), that suggests that serial mating (leaving one relationship and entering another one) led humans to anticipate and appraise opportunities to mate-switch. According to this theory, humans monitor potential alternatives to their current partner and cultivate “buck-up mates,” should their current relationship fail (Buss et al., 2017). Thus, the authors of this study propose an additional item to the reasons to engage in sex. The question could be formulated in the following way: “I engaged in sex as I wanted to have a backup relationship in case things go wrong with my current partner.”

It is important to mention that procreation as a reason to engage in sex is somewhat problematic in the context of this study. While there is a significant difference to engage in sex for this reason with one’s primary and secondary partners, these numbers may not reflect the actual state of things correctly as there was a number of gay/lesbian and bisexual people in the sample. On top of that it should be assumed that a portion of respondents may be infertile, past reproductive age or not willing to have children, which may affect the ranking of the reason compared to other reasons to engage in sex.

Different relationships may have different functions in one’s life and thus lead to differences in motivation to engage in sex with various partners. However, to get a broader understanding of sexual motivation, substantially more research should be performed that considers nuances of non-monogamous relationship structures, looking separately at each of the relationship arrangements. Also, additional exploration how sexual motivation is related to sexual orientation should be explored.

## Data availability statement

The raw data supporting the conclusions of this article will be made available by the authors, without undue reservation.

## Ethics statement

The studies involving human participants were reviewed and approved by the Ethics Committee for Humanities and Social Sciences research involving human participants, University of Latvia. The patients/participants provided their written informed consent to participate in this study.

## Author contributions

AK conceptualized and designed the study, organized the database, performed the statistical analysis, and wrote the first draft and sections of the manuscript. All authors contributed to the article and approved the submitted version.

## Conflict of interest

The authors declare that the research was conducted in the absence of any commercial or financial relationships that could be construed as a potential conflict of interest.

## Publisher’s note

All claims expressed in this article are solely those of the authors and do not necessarily represent those of their affiliated organizations, or those of the publisher, the editors and the reviewers. Any product that may be evaluated in this article, or claim that may be made by its manufacturer, is not guaranteed or endorsed by the publisher.
